# The Role of Immunotherapy in the Management of Esophageal Cancer in Patients Treated with Neoadjuvant Chemoradiation: An Analysis of the National Cancer Database

**DOI:** 10.3390/cancers16132460

**Published:** 2024-07-04

**Authors:** Panagiotis Tasoudis, Vasiliki Manaki, Yoshiko Iwai, Steven A. Buckeridge, Audrey L. Khoury, Chris B. Agala, Benjamin E. Haithcock, Gita N. Mody, Jason M. Long

**Affiliations:** 1 Division of Cardiothoracic Surgery, Department of Surgery, School of Medicine, University of North Carolina at Chapel Hill, Chapel Hill, NC 27514, USA; yoshiko_iwai@med.unc.edu (Y.I.); steven.buckeridge@unchealth.unc.edu (S.A.B.); audrey.khoury@unchealth.unc.edu (A.L.K.); benjamin_haithcock@med.unc.edu (B.E.H.); gita_mody@med.unc.edu (G.N.M.); jason_long@med.unc.edu (J.M.L.); 2 School of Medicine, Aristotle University of Thessaloniki, 54124 Thessaloniki, Greece; vassiamanaki@gmail.com; 3 Department of Surgery, School of Medicine, University of North Carolina at Chapel Hill, Chapel Hill, NC 27514, USA; chris_agala@med.unc.edu

**Keywords:** esophageal cancer, immunotherapy, adjuvant immunotherapy, neoadjuvant immunotherapy

## Abstract

**Simple Summary:**

Esophageal cancer is a severe disease with a high mortality rate. Recent advancements in treatments have improved outcomes, but survival rates remain low. This study investigates the use of immunotherapy, a treatment that helps the immune system fight cancer, in combination with standard chemoradiation and surgery for patients with locally advanced esophageal cancer. By analyzing data from the National Cancer Database, we found that patients who received adjuvant immunotherapy had better survival rates than those who did not receive immunotherapy or who received neoadjuvant immunotherapy. These findings suggest that adjuvant immunotherapy could be a beneficial addition to existing treatment protocols, and further research is needed to confirm these results and optimize patient outcomes.

**Abstract:**

Background: The current National Comprehensive Cancer Network advises neoadjuvant chemoradiotherapy followed by surgery for locally advanced cases of esophageal cancer. The role of immunotherapy in this context is under heavy investigation. Methods: Patients with esophageal adenocarcinoma were identified in the National Cancer Database (NCDB) from 2004 to 2019. Three groups were generated as follows: (a) no immunotherapy, (b) neoadjuvant immunotherapy, and (c) adjuvant immunotherapy. Overall survival was evaluated using the Kaplan–Meier method and Cox proportional hazard analysis, adjusting for previously described risk factors for mortality. Results: Of the total 14,244 patients diagnosed with esophageal adenocarcinoma who received neoadjuvant chemoradiation, 14,065 patients did not receive immunotherapy, 110 received neoadjuvant immunotherapy, and 69 received adjuvant immunotherapy. When adjusting for established risk factors, adjuvant immunotherapy was associated with significantly improved survival compared to no immunotherapy and neoadjuvant immunotherapy during a median follow-up period of 35.2 months. No difference was noted among patients who received no immunotherapy vs. neoadjuvant immunotherapy in the same model. Conclusions: In this retrospective analysis of the NCDB, receiving adjuvant immunotherapy offered a significant survival advantage compared to no immunotherapy and neoadjuvant immunotherapy in the treatment of esophageal adenocarcinoma. The addition of neoadjuvant immunotherapy to patients treated with neoadjuvant chemoradiation did not improve survival in this cohort. Further studies are warranted to investigate the long-term outcomes of immunotherapy in esophageal cancer.

## 1. Introduction

Esophageal cancer is attributable for more than half a million cancer-related deaths worldwide each year [1,2]. Esophageal cancer is traditionally linked with poor prognosis regardless of the histological subtype; however, advances in treatment options over the past few years have led to improved outcomes [3]. Surgical resection has been the standard of care for early-stage esophageal cancer [4]. Research on patients who have undergone neoadjuvant chemoradiation shows encouraging outcomes [4,5,6,7,8] and based on these reports, the updated National Comprehensive Cancer Network guidelines recommend neoadjuvant chemoradiotherapy followed by surgical resection in cases of locally advanced disease [6]. 

The emergence of immunotherapy has led to a paradigm shift in medical oncology and the role of immunotherapy in the treatment of esophageal cancer has been heavily investigated in recent years [9]. The National Cancer Database (NCDB) captures the majority of newly diagnosed esophageal cancer cases in the United States (US). In this study, we assessed recent trends in the use of immunotherapy as an adjuvant versus neoadjuvant therapy for locally advanced esophageal cancer treated with neoadjuvant chemoradiotherapy followed by surgical resection in the NCDB.

## 2. Materials and Methods

A retrospective analysis using the 2020 version of the National Cancer Database (NCDB) was performed, which included patients from 2004 to 2019. The NCDB is a hospital-based tumor registry run jointly by the Commission on Cancer of the American College of Surgeons and the American Cancer Society that captures the majority of newly diagnosed esophageal cancer cases in the US. In accordance with the NCDB Data Use Agreement, the American College of Surgeons and the Commission on Cancer have not verified and are not responsible for the analytic or statistical methodology employed, or the conclusions drawn from these data by the investigators.

The NCDB was queried for adults at least 18 years of age diagnosed with esophageal cancer who received neoadjuvant chemoradiotherapy prior to surgery between 2004 and 2019. Three groups were generated based on the timing of immunotherapy administration: (a) a “no immunotherapy” group that included patients who did not receive any immunotherapy, (b) a “neoadjuvant immunotherapy” group that included patients who received immunotherapy 120 days prior to their primary surgery, and (c) an “adjuvant immunotherapy” group that included patients who received immunotherapy within 120 days of their primary surgery. To diminish the risk of bias, patients were excluded if they had histology other than adenocarcinoma, if they had positive surgical margins, if surgical margins were not available, or if they underwent local tumor excision, esophagectomy with laryngectomy, or esophagectomy with total gastrectomy. All definitions regarding the characteristics of the patients and treatment plans were identified using the Systemic Surgery Sequence and Radiation Surgery Sequence variables.

The Institutional Review Board (IRB) at the University of North Carolina in compliance with the Health Insurance Portability and Accountability Act of 1996 regulations and the Declaration of Helsinki approved this study (#20-1493). Patient consent was not required given the nature of this retrospective study. The data used in the study were derived from a deidentified NCDB file.

### Statistical Analysis

Patients’ baseline characteristics, tumor characteristics, treatment details, and clinical outcomes data were expressed as frequencies with corresponding percentages for categorical variables and as medians [first quartile, third quartile] for continuous data.

The Kaplan–Meier method was used to calculate and plot overall survival (OS), using deidentified time-to-event data. Of note, the time of diagnosis was regarded as the timepoint zero for our cohort. Adjusted and unadjusted multivariate Cox proportional hazard models were constructed using covariates determined a priori to be of prognostic importance in this patient population: age, sex, presence of comorbidities, disease stage, lymph node status, and administration of immunotherapy.

## 3. Results

### 3.1. Patient Baseline and Tumor Characteristics

We identified 14,244 patients with esophageal adenocarcinoma who received neoadjuvant chemoradiation between 2004 and 2019 in the NCDB (Table 1). A total of 14,065 patients did not receive any form of immunotherapy, 110 patients received neoadjuvant immunotherapy, and 69 patients received adjuvant immunotherapy. In all three groups, the majority of patients were male (88.6%) and White (96.7%). In the “no immunotherapy” cohort, 72.7% of the identified patients had a Charlson–Deyo score of zero, whereas in the “neoadjuvant immunotherapy” and “adjuvant immunotherapy” groups, the respective percentages were 71.8% and 79.7%. There were no significant differences in the breakdown of Charlson–Deyo scores among patients receiving no immunotherapy vs. neoadjuvant therapy vs. adjuvant therapy (*p* = 0.47). There were significant differences appreciated for year of diagnosis (*p* < 0.001), type of insurance (*p* = 0.032), and median income quartile (*p* = 0.002) when comparing across the three patient groups.

Regarding tumor characteristics, stage III tumors were most common across the groups at 40.2% in the “no immunotherapy” group, 52.7% in the “neoadjuvant immunotherapy” group, and 50.7% in the “adjuvant immunotherapy” group (Table 2). The “no immunotherapy” group also had a relatively high proportion of patients with stage II tumors at 35.3%, compared to the “neoadjuvant immunotherapy” (20.9%) and “adjuvant immunotherapy” (21.7%) groups. There were statistically significant differences appreciated in the distribution of tumor stages across the three patient groups (*p* < 0.001). A total of 36.1% of the included patients in the “no immunotherapy” cohort had positive pathologic lymph nodes, whereas 42.7% and 73.9% of patients had positive lymph nodes in the “neoadjuvant immunotherapy” and “adjuvant immunotherapy” cohorts, respectively (all *p* < 0.001; Table 2). Tumor grade was similar across the three groups (*p* = 0.12) with most patients documented as having either moderately differentiated or poorly differentiated tumors.

In terms of treatment facility, the majority of patients were treated in an academic hospital for the “no immunotherapy” cohort (46.3%), the “neoadjuvant immunotherapy” cohort (51.4%), and the “adjuvant immunotherapy” cohort (46.9%; Table 2).

### 3.2. Overall Survival 

**Figure 1:**
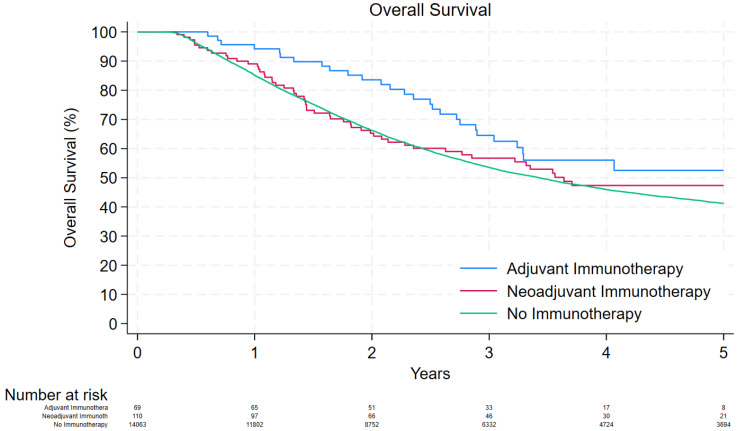
Kaplan–Meier curve depicting the overall survival of patients that were treated with adjuvant immunotherapy, neoadjuvant immunotherapy, and no immunotherapy.

The median follow-up time in the “no immunotherapy” cohort was 31.9 [interquartile range (IQR): 17.0, 62.5] months and the 1- and 5-year OS was 85.1% and 41.2%, respectively. The median follow-up times in the “neoadjuvant immunotherapy” and “adjuvant immunotherapy” groups were 30.6 [16.3, 53.9] and 35.2 [23.8, 47.4], respectively. The 1- and 5-year OS was 89.1% and 47.4% in the “neoadjuvant immunotherapy” cohort and 94.2% and 52.5% in the “adjuvant immunotherapy” cohort, respectively (Figure 1).

Receiving adjuvant immunotherapy was associated with better survival outcomes compared to receiving no immunotherapy (unadjusted hazard ratio [HR]: 0.66, 95% Confidence Interval [CI]: 0.45–0.96, *p* = 0.032; adjusted HR: 0.65, 95% CI: 0.44–0.94, *p* = 0.001). However, receiving neoadjuvant immunotherapy had similar survival outcomes as receiving no immunotherapy (unadjusted HR: 0.86, 95% CI: 0.65–1.13, *p* = 0.28; adjusted HR: 0.82, 95% CI: 0.63–1.08, *p* = 0.25). Other factors that were associated with worse survival outcomes were increased age (*p* < 0.001), male gender (*p* < 0.001), Charlson–Deyo comorbidity score ≥1 (*p* < 0.001), and high disease stage (*p* < 0.001; Table 3).

## 4. Discussion

The findings of this study suggest that receiving adjuvant immunotherapy may be beneficial for overall survival in patients with esophageal cancer treated with guideline-concordant neoadjuvant chemoradiotherapy. Interestingly, receiving neoadjuvant immunotherapy was associated with equivalent overall survival as patients who received no immunotherapy, even when adjusting for age, gender, comorbidities, and disease stage. Similarly, age, male gender, higher comorbidity score, and higher disease stage were all found to be associated with worse survival results. 

Overall, our findings are consistent with previous reports regarding immunotherapy in the setting of esophageal cancer [10,11]. Since the release of the landmark CROSS trial from the Dutch Cancer Foundation in 2012, neoadjuvant chemoradiotherapy followed by surgical resection remains the gold standard for treating esophageal or gastroesophageal junction (GEJ) malignancies [9]. Long-term survival, however, does not surpass 50% five years after surgery [4,5,6]. With evidence supporting the efficacy of neoadjuvant chemoradiation in the setting of esophageal cancer, we aimed to assess the safety and efficacy of neoadjuvant immunotherapy in patients who received guideline-concordant neoadjuvant chemoradiation. 

The use of adjuvant immunotherapy in the treatment of esophageal cancers has been demonstrated to be effective. The CheckMate 577 trial investigated the impact of the checkpoint inhibitor nivolumab as an adjuvant therapy in patients with pathologic residual esophageal malignancy after neoadjuvant chemoradiation [11]. In this clinical trial, adjuvant immunotherapy with nivolumab enhanced median disease-free survival (22.4 months) compared to the placebo group (11 months) [11]. Our findings are consistent with the findings of the CheckMate 577 trial, which indicated that adjuvant immunotherapy was associated with better survival than receiving no immunotherapy, where improved survival may be attributed to lower cancer recurrence rates. It must be stated, however, that esophagectomy is associated with major morbidity and a prolonged recovery period, which can delay the initiation of any adjuvant therapies. In addition, immunotherapy may impede recovery from surgery and cause adverse outcomes in some patients, as seen in the ChekMate 577 trial where total adverse event rates (about 30%) were similar in the placebo and nivolumab groups [11]. Even though nivolumab increased disease-free survival in multiple subgroups, it is important to highlight that the study was discontinued due to major adverse events associated with the active drug in 9% of the nivolumab group and 3% of the placebo group [11]. 

Neoadjuvant immunotherapy has the potential to contribute to disease downstaging in locally advanced cancers, improving R0 resection rates in addition to improved major or complete pathologic responses [12,13,14]. Postoperative recovery may also be less likely to be prolonged with immunotherapy given prior to surgical resection [12,15]. However, preoperative immunomodulation may increase the likelihood of intra- and postoperative complications [16]. Unfortunately, strong evidence is lacking on this topic. In a retrospective study by Sihag et al., neoadjuvant immunotherapy was reported as being safe and effective in the early postoperative period; however, the number of patients in the study was small, and no long-term outcomes were reported [17]. A relevant meta-analysis assessing the efficacy of neoadjuvant immunotherapy combined with chemotherapy in resectable esophageal cancer reported high rates of both major and complete pathological response; however, the authors did not assess long-term outcomes. In our study, there was no statistically significant difference in long-term overall survival among patients receiving neoadjuvant immunotherapy vs. no immunotherapy at all.

Of note, several clinical studies in the literature have reported on the effects of neoadjuvant and adjuvant immunotherapy in esophageal cancer [9,13,18,19] and there are several ongoing clinical trials that aim to clarify the role of immunotherapy in the setting of esophageal cancer. For example, the ECOG-ACRIN Cancer Research Group Trial (EA2174) is comparing outcomes associated with receiving neoadjuvant vs. adjuvant immunotherapy in patients with locoregional esophageal or GEJ cancer. In addition, immunotherapy is being administered as adjuvant therapy in ATTRACTION-5, a randomized, multicenter, double-blind, placebo-controlled phase III nivolumab study. Finally, KEYNOTE-585 is investigating chemotherapy with or without pembrolizumab in patients with gastric cancer, KEYNOTE-975 is evaluating definitive chemoradiotherapy plus pembrolizumab in patients with esophageal tumors, and KEYNOTE-859 is examining the use of immunotherapy in metastatic esophageal cancer.

There are some limitations of this study that should be acknowledged. To begin with, the extent of downstaging could not be estimated in the NCDB and thus we could not assess the impact of neoadjuvant immunotherapy in that regard. Additionally, the combined positive score (or CPS score), which is used to assess PD-(L) 1 status and predict immunotherapy response, was unavailable in the NCDB and this may have confounded some of our findings. It is important to note that the NCDB does not capture detailed information on specific chemotherapy protocols (e.g., FLOT or DOC) or the types of immunotherapy administered, which limits our ability to analyze the impact of these specific treatment regimens on patient outcomes [20,21]. Furthermore, disease recurrence, intra- and postoperative morbidity following adjuvant and neoadjuvant immunotherapy were not mentioned in the NCDB. The relatively small numbers of patients receiving neoadjuvant and adjuvant immunotherapy imposed constraints on the statistical power. The majority of patients were identified as White [17] and there was a male gender predominance, which is consistent with the literature [22]. Therefore, caution is necessary when interpreting our findings, especially for a more demographically diverse patient population. It is also important to acknowledge that our patient cohort predates the release of the CheckMate 577 trial results and neither surgeon nor oncologist intent are captured in the NCDB. However, given that the NCDB includes approximately 70% of all cancer cases in the US, and considering the trial‘s focus on academic centers, it is plausible that some patients within our cohort may have been included in the CheckMate 577 trial.

## 5. Conclusions

In this retrospective analysis of the NCDB, patients with esophageal adenocarcinoma who were treated with adjuvant immunotherapy had a significant survival advantage over patients who received no immunotherapy or neoadjuvant immunotherapy. Therefore, the addition of neoadjuvant immunotherapy to neoadjuvant chemoradiation did not appear to improve survival in this cohort. Appropriate patient selection and identifying and overcoming underlying mechanisms of primary immunotherapy resistance are all key challenges that should be addressed in future research. Further investigation of the long-term results of adjuvant and neoadjuvant immunotherapy in esophageal cancer is warranted from high-quality prospective clinical studies.

## Figures and Tables

**Table 1:** Baseline characteristics of patients diagnosed with esophageal adenocarcinoma in the National Cancer Database, 2004–2019.

	No Immunotherapy, No. (%)	Neoadjuvant Immunotherapy, No. (%)	Adjuvant Immunotherapy, No. (%)	*p*-Value
	N = 14,065	N = 110	N = 69	
Age (Median, (IQR)) *	56 (63, 69)	62 (55, 67)	59 (53, 66)	<0.001
**Gender**				0.164
Male	12,453 (88.54)	96 (87.27)	66 (95.65)	
Female	1612 (11.46)	14 (12.73)	3 (4.35)	
**Race**				0.688
White	13,605 (96.73)	106 (96.36)	69 (100)	
African American	189 (1.34)	1 (0.91)	0 (0)	
Other	271 (1.93)	3 (2.73)	0 (0)	
**Year of diagnosis**				<0.0001
2004	541 (3.85)	0 (0)	1 (1.45)	
2005	578 (4.11)	0 (0)	0 (0)	
2006	640 (4.55)	0 (0)	0 (0)	
2007	650 (4.62)	0 (0)	0 (0)	
2008	728 (5.18)	1 (0.91)	0 (0)	
2009	724 (5.15)	0 (0)	0 (0)	
2010	665 (4.73)	0 (0)	0 (0)	
2011	793 (5.64)	0 (0)	0 (0)	
2012	886 (6.3)	1 (0.91)	1 (1.45)	
2013	984 (7)	8 (7.27)	2 (2.9)	
2014	1051 (7.47)	18 (16.36)	4 (5.8)	
2015	1107 (7.87)	12 (10.91)	0 (0)	
2016	1109 (7.88)	11 (10)	10 (14.49)	
2017	1131 (8.04)	21 (19.09)	20 (28.99)	
2018	1257 (8.94)	22 (20)	24 (34.78)	
2019	1221 (8.68)	16 (14.55)	7 (10.14)	
**Type of insurance**				0.032
Not Insured	256 (1.82)	5 (4.55)	0 (0)	
Private Insurance/ Managed Care	6992 (49.71)	55 (50)	43 (62.32)	
Medicaid	627 (4.46)	4 (3.64)	5 (7.25)	
Medicare	5753 (40.9)	39 (35.45)	19 (27.54)	
Other Government		242 (1.72)	5 (4.55)	2 (2.9)
Unknown	195 (1.39)	2 (1.82)	0 (0)	
**Median Income Quartiles, 2012–2016**				0.002
<$40,227	1679 (11.9)	11 (10)	8 (11.6)	
$40,227–$50,353	2837 (20.2)	12 (10.9)	10 (14.5)	
$50,354–$63,332	3128 (22.2)	16 (14.5)	14 (20.3)	
≥$63,333	4429 (31.5)	52 (47.3)	29 (42)	
Not applicable	5797 (41.2)	18 (16.4)	22 (31.9)	
**Charlson–Deyo Score**				0.473
0	10222 (72.68)	79 (71.82)	55 (79.71)	
1	2847 (20.24)	20 (18.18)	8 (11.59)	
2	689 (4.9)	8 (7.27)	5 (7.25)	
3	307 (2.18)	3 (2.73)	1 (1.45)	

All values are reported as frequencies (corresponding %) or medians [first quartile, third quartile]. * IQR = interquartile range.

**Table 2:** Clinical characteristics of patients diagnosed with esophageal adenocarcinoma in the National Cancer Database, 2004–2019.

	No Immunotherapy, No. (%)	Neoadjuvant Immunotherapy, No. (%)	Adjuvant Immunotherapy, No. (%)	*p*-Value
**AJCC stage group**				<0.001
I	2131 (15.15)	12 (10.91)	3 (4.35)	
II	4965 (35.3)	23 (20.91)	15 (21.74)	
III	5648 (40.16)	58 (52.73)	35 (50.72)	
IV	719 (5.11)	8 (7.27)	7 (10.14)	
Not applicable	602 (4.28)	9 (8.18)	9 (13.04)	
**Lymph Nodes**				<0.001
Negative	7845 (55.78)	56 (50.91)	18 (26.09)	
Positive	5082 (36.13)	47 (42.73)	51 (73.91)	
Not assessed	1138 (8.09)	7 (6.36)	0 (0)	
**Grade**				0.159
Well differentiated	422 (3.64)	1 (1.39)	1 (2.63)	
Moderately differentiated	4115 (35.51)	38 (52.78)	12 (31.58)	
Poorly differentiated	5286 (45.62)	23 (31.94)	19 (50)	
Undifferentiated	145 (1.25)	0 (0)	0 (0)	
Not applicable	1619 (13.97)	10 (13.89)	6 (15.79)	
**Facility type**				0.262
Community Cancer Program	567 (4.09)	7 (6.54)	4 (6.25)	
Comprehensive Community Cancer Program	4224 (30.48)	24 (22.43)	14 (21.88)	
Academic/Research Program	6422 (46.34)	55 (51.4)	30 (46.88)	
Integrated Network Cancer Program	2644 (19.08)	21 (19.63)	16 (25)	
**Days from radiation to surgery, Median (IQR)**	89 (79, 100)	88 (78, 102)	83 (77, 97)	0.514
**Days from chemotherapy to surgery, Median (IQR)**	90 (79, 100)	92 (79, 105)	84 (77, 98)	0.937
**Follow-up,** **Median (IQR)**	31.9 (16.95, 62.49)	30.63 (16.26, 53.91)	35.15 (23.79, 47.41)	0.022
**Readmission within 30 days, Median (IQR)**	723 (5.14)	9 (8.18)	2 (2.9)	0.737
**Length of hospital stay, Median (IQR)**	9 (7, 13)	8 (9, 14)	8 (7, 11)	0.062

Abbreviations: NOS: not otherwise specified; AJCC: American Joint Committee on Cancer. All values are reported as frequencies (corresponding %) or medians [first quartile, third quartile] as specified.

**Table 3:** Survival outcomes of patients diagnosed with esophageal adenocarcinoma in the National Cancer Database, 2004–2019.

	Estimates Adjusted Based on Immunotherapy	95% CI			Adjusted Estimates	95% CI		
Characteristic	HR	LCL	UCL	*p*-Value	HR	LCL	UCL	*p*-Value
**Immunotherapy**								
None	reference	reference	reference	reference	reference	reference	reference	reference
Neoadjuvant	0.86	0.65	1.13	0.278	0.82	0.63	1.08	0.249
Adjuvant	0.66	0.45	0.96	0.032	0.65	0.44	0.94	0.001
Neoadjuvant vs. adjuvant	0.73	0.46	1.16	0.179	0.65	0.40	1.06	0.083
**Age**	1.02	1.01	1.02	<0.001	1.02	1.01	1.02	<0.001
**Gender**								
Male	reference	reference	reference	reference	reference	reference	reference	reference
Female	0.86	0.80	0.92	<0.001	0.86	0.81	0.93	<0.001
**Charlson** **–** **Deyo Score**								
0	reference	reference	reference	reference	reference	reference	reference	reference
1	1.16	1.10	1.22	<0.001	1.15	1.09	1.21	<0.001
2	1.23	1.12	1.35	<0.001	1.18	1.08	1.30	<0.001
3	1.42	1.23	1.63	<0.001	1.35	1.17	1.55	<0.001
**AJCC stage group**								
I	reference	reference	reference	reference	reference	reference	reference	reference
II	1.32	1.23	1.41	<0.001	1.33	1.24	1.42	<0.001
III	1.68	1.57	1.79	<0.001	1.70	1.59	1.82	<0.001
IV	1.82	1.63	2.03	<0.001	1.94	1.74	2.16	<0.001
Not applicable	1.61	1.43	1.81	<0.001	1.66	1.48	1.87	<0.001

## Data Availability

The data underlying this article are available in the article.
